# The Marine Sponge-Derived Inorganic Polymers, Biosilica and Polyphosphate, as Morphogenetically Active Matrices/Scaffolds for the Differentiation of Human Multipotent Stromal Cells: Potential Application in 3D Printing and Distraction Osteogenesis

**DOI:** 10.3390/md12021131

**Published:** 2014-02-21

**Authors:** Xiaohong Wang, Heinz C. Schröder, Vladislav Grebenjuk, Bärbel Diehl-Seifert, Volker Mailänder, Renate Steffen, Ute Schloßmacher, Werner E. G. Müller

**Affiliations:** 1 ERC Advanced Investigator Grant Research Group, Institute for Physiological Chemistry, University Medical Center, Johannes Gutenberg University, Duesbergweg 6, D-55128 Mainz, Germany; E-Mails: hschroed@uni-mainz.de (H.C.S.); grebenyu@uni-mainz.de (V.G.); steffen@uni-mainz.de (R.S.); schlossm@uni-mainz.de (U.S.); 2 NanotecMARIN GmbH, 55128 Mainz, Germany; E-Mail: helmut-baerbel.Seifert@t-online.de; 3 Max Planck Institute for Polymer Research, Ackermannweg 10, 55129 Mainz, Germany; E-Mail: volker.mailaender@mpip-mainz.mpg.de; 4 Medical Clinic, University Medical Center, Johannes Gutenberg University, Langenbeckstr. 1, D-55131 Mainz, Germany

**Keywords:** biosilica, polyphosphate, multipotent stromal cells, mesenchymal stem cells, alkaline phosphatase, 3D cell/tissue printing, distraction osteogenesis

## Abstract

The two marine inorganic polymers, biosilica (BS), enzymatically synthesized from ortho-silicate, and polyphosphate (polyP), a likewise enzymatically synthesized polymer consisting of 10 to >100 phosphate residues linked by high-energy phosphoanhydride bonds, have previously been shown to display a morphogenetic effect on osteoblasts. In the present study, the effect of these polymers on the differential differentiation of human multipotent stromal cells (hMSC), mesenchymal stem cells, that had been encapsulated into beads of the biocompatible plant polymer alginate, was studied. The differentiation of the hMSCs in the alginate beads was directed either to the osteogenic cell lineage by exposure to an osteogenic medium (mineralization activation cocktail; differentiation into osteoblasts) or to the chondrogenic cell lineage by incubating in chondrocyte differentiation medium (triggering chondrocyte maturation). Both biosilica and polyP, applied as Ca^2+^ salts, were found to induce an increased mineralization in osteogenic cells; these inorganic polymers display also morphogenetic potential. The effects were substantiated by gene expression studies, which revealed that biosilica and polyP strongly and significantly increase the expression of bone morphogenetic protein 2 (BMP-2) and alkaline phosphatase (ALP) in osteogenic cells, which was significantly more pronounced in osteogenic *versus* chondrogenic cells. A differential effect of the two polymers was seen on the expression of the two collagen types, I and II. While collagen Type I is highly expressed in osteogenic cells, but not in chondrogenic cells after exposure to biosilica or polyP, the upregulation of the steady-state level of collagen Type II transcripts in chondrogenic cells is comparably stronger than in osteogenic cells. It is concluded that the two polymers, biosilica and polyP, are morphogenetically active additives for the otherwise biologically inert alginate polymer. It is proposed that alginate, supplemented with polyP and/or biosilica, is a suitable biomaterial that promotes the growth and differentiation of hMSCs and might be beneficial for application in 3D tissue printing of hMSCs and for the delivery of hMSCs in fractures, surgically created during distraction osteogenesis.

## 1. Introduction

Bone formation is a complex process involving several cell lineages and growth factors, as well as an ordered scaffold, comprising a fibrillar organic network. The major categories of bone cells are the bone forming osteoblasts and the bone resorbing osteoclasts. In addition, osteocytes are found in mature bone, which do not divide and are derived from osteoprogenitors. Finally, lining cells cover the bone surface. During bone formation, a controlled cross-talk and coupling between the major cells takes place that maintains the balance between the anabolic osteoblasts and the catabolic osteoclasts [1]. While human osteoblasts derive from multipotent stromal cells (hMSC), previously also termed mesenchymal stem cells, osteoclasts differentiate from the monocyte/macrophage hematopoietic lineage and develop and adhere onto bone matrix.

Human MSCs (hMSCs), discovered in 1968 by Friedenstein [2], have the capacity to readily differentiate into the osteogenic, chondrogenic, adipogenic or myogenic cell lineage, depending on the activation of specific transcription factors. They can be efficiently expanded in culture; even after million-fold expansion, they retain the ability to differentiate (see [3]). Since hMSCs are very contact inhibited, they need to be expanded *ex vivo*. hMSCs are considered to be very promising candidates for bone and cartilage regeneration [4], due to their high osteogenic differentiation capacity. A promising approach to accelerate the healing of bone defects, including fractures created during bone lengthening by distraction osteogenesis (DO), might be the delivery of autologous MSCs directly to the damaged site [5]. This approach would provide a new strategy to manage the major, well-known problems caused by the common treatment of bone defects by autogenic bone grafting (e.g., [6]). At present, the most promising method for the application of MSCs appears to be the injection/delivery of the cells, embedded into platelet lysate from whole blood-derived pooled platelet concentrates and apheresis-derived platelet concentrates for the isolation and expansion of human bone marrow mesenchymal stromal cells [7]. Even though this technique sounds straightforward, it is presently difficult to deliver the cells to the desired location. Furthermore, a suitable, more solid-state matrix for embedding the MSCs during the injection process would improve the desired outcome.

Likewise important is the composition of a morphogenetically active scaffold for the three-dimensional (3D) growth of MSCs. A solution for formulating a suitable scaffold for MSCs would provide the basis for a future 3D printing/bioprinting of cells and human tissue/organs [8]. As a first step to reach this goal, the application of 3D printing in prosthetics, for the fabrication of human tissue bioprints inserted into lesions created during DO, is proposed. DO is an established and widely used technique for regenerating endogenous bone in orthopedic and maxillofacial surgery [9]. This surgical procedure aims to elicit a controlled regenerative process by the application of an active mechanical strain in order to initiate and enhance the biological response in the injured tissues to form new bone tissue. The regeneration process during DO includes four distinct stages [10]: (i) osteotomy; (ii) the latency phase, comprising the period between osteotomy and distraction (during this period, soft callus is formed); (iii) the distraction phase, during which traction is applied to transport bone; the formation of new immature woven and parallel-fibered bone islands commences; (iv) the consolidation phase, during which maturation and corticalization of the regenerating bone occurs. During the distraction phase, a dynamic microenvironment is established, characterized by an increased angiogenesis, which is paralleled and followed by an increased proliferation of spindle-shaped fibroblasts. In turn, collagen (mostly Type I) is formed alongside the angiogenic centers, allowing intramembranous, but not endochondral, ossification [11]. Besides an intense osteoblastic differentiation activity [12], occasionally, also, trans-differentiation processes of chondroblasts, as well as of fibroblasts into osteoblasts have been reported. During the distraction phase, as well as during the consolidation phase, the high expression of the genes encoding for the bone morphogenetic proteins 2 and 4 (BMP-2 and BMP-4) has been described [13]. A series of complications associated with DO has been reported that, besides intra-operative difficulties, include, in particular, intra-distraction complications, which arise during distraction, as well as post-distraction complications, which concern the late problems arising during the period of splinting, e.g., malunion or relapse [14].

Recently, we developed a morphogenetically active scaffold, based on biosilica-alginate hydrogel [8,15]. Alginates, polysaccharides, allow the fabrication of a variety of biomaterials suitable for tissue engineering, e.g., gels and fibers, and are suitable vehicles for injectable solutions as pastes [16]. If those alginates are enriched with biosilica, the fabricated hydrogel has been shown to provide a morphogenetically active scaffold for bone-related SaOS-2 cells *in vitro* [8]. Biosilica is a naturally occurring polymer used by the oldest metazoans, the sponges (phylum: Porifera), as elements for their spicule formation (reviewed in [17,18]). A likewise polymeric inorganic material is polyphosphate (polyP), which occurs in any living organisms and at high concentrations in sponges, as well (see [17]).

Based on initial studies [19,20], we discovered that biosilica, enzymatically formed from ortho-silicate by the enzyme silicatein [18], displays an inductive anabolic bone-forming effect on SaOS-2 cells. This polymer causes a significant shift of the OPG-RANKL (osteoprotegerin: receptor activator of nuclear factor-κB ligand) ratio [21], resulting in an inhibition of the differentiation pathway of pre-osteoclasts into mature osteoclasts. In addition to an increased mineralization, biosilica has been shown to increase the expression of BMP-2 in SaOS-2 cells [22]. Finally, biosilica shows osteogenic potential [21]. These data have been supported recently [23] using hMSCs.

PolyP is known to act as a storage substance of energy, a chelator for metal cations, a phosphate donor for sugars and adenylate kinase and an inducer of apoptosis; in addition, it is involved in mineralization processes of bone tissue (reviewed in [17]). Moreover, polyP acts as a modulator of gene expression, e.g., in the osteoblast-like cell lines, MC3T3-E1 and SaOS-2 cells, and in hMSCs, and causes an increased expression of the genes encoding for osteocalcin, osterix, bone sialoprotein, BMP-2 and tissue nonspecific alkaline phosphatase, all proteins that are crucial for bone formation ([15]; reviewed in [24]).

**Figure 1:**
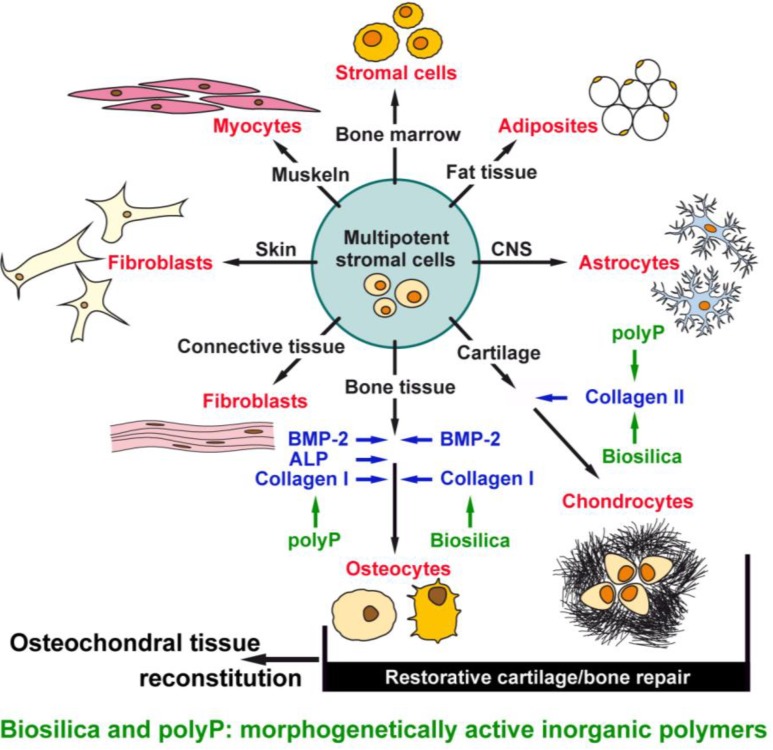
Multipotent differentiation of human multipotent stromal cells (hMSC). Specific transcription factors determine both the commitment and the differentiation of hMSCs towards the osteogenic, chondrogenic, adipogenic or myogenic lineage. The osteogenic and the chondrogenic lineages are involved in the restorative repair of bone and cartilage tissue (osteochondral tissue reconstitution). Biosilica and polyphosphate (polyP) display anabolic, morphogenetic effects on those two differentiation lines.

The available data indicate that both SaOS-2 cells and hMSCs, after encapsulation into alginate hydrogels, can retain their proliferation and differentiation-promoting activity if the matrix had been supplemented with biosilica and polyP. hMSCs can differentiate into several lineages (Figure 1), dependent on the inducers added to the assay system [25]. Osteogenic differentiation is triggered by incubation in medium/fetal calf serum (FCS), supplemented with dexamethasone, ascorbic acid and sodium β-glycerophosphate. Chondrogenic differentiation occurs in medium/serum, supplemented with transforming growth factor-β1, insulin, transferrin, dexamethasone and ascorbic acid. Adipogenic differentiation is promoted by medium/FCS, indomethacin, dexamethasone and 3-isobutyl-1-methylxanthine and insulin. Neurogenic differentiation is favored if the cells are incubated with β-mercaptoethanol.

The hMSCs provide a suitable cell source for osteochondral tissue reconstruction [26], required for an acceleration of the ossification processes during OD or after the transplantation of 3D tissue-like implants (Figure 1). Figure 1 also highlights recent findings that biosilica and polyP acts in an organic scaffold, like alginate, as a morphogenetically active inorganic polymer.

In the present study, we studied the differentiation of hMSCs towards the osteocyte and chondrocyte lineages. Both cell lineages are involved in bone formation and cartilage repair, two processes intimately involved in bone growth [27]. In turn, hMSCs are, due to their osteogenic and chondrogenic potential, attractive candidates for restorative cartilage/bone repair. Chondrocytes are known to express high levels of collagen Type II, while osteoblasts express collagen Type I [28]. Here, we used hMSCs to elucidate the morphogenetic potential of the two polymers of marine origin, biosilica and polyP, with respect to their differentiation capacity on the osteogenic differentiation, as well as on the chondrogenic differentiation lineage. The data show that these two polymers display a morphogenetic effect on both cell lineages.

## 2. Results and Discussion

### 2.1. Cultivation of hMSCs and Encapsulation into Alginate Beads

**Figure 2:**
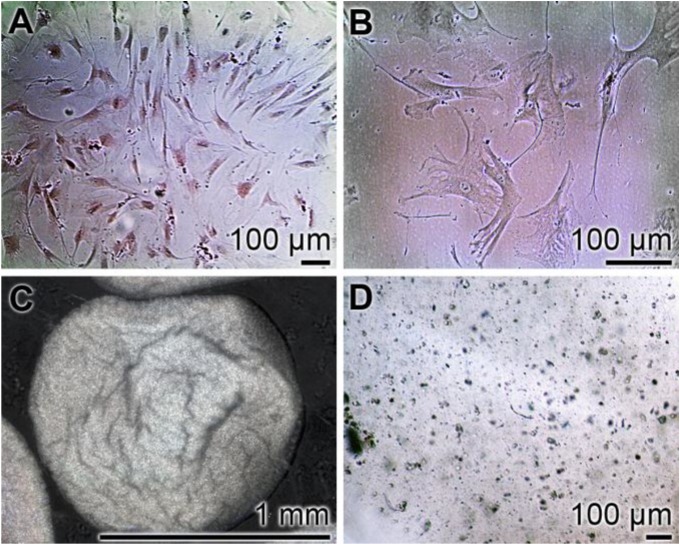
Light microscopic images of hMSCs, cultured in a monolayer (**A** and **B**), adherent to the plastic surface. The hMSCs induced to the osteogenic or the chondrogenic lineages were embedded into alginate beads (**C** and **D**).

hMSCs were incubated in an α-MEM/FCS medium, as described in “Experimental Section”. They show the characteristic plastic adherent properties (higher passage number) (Figure 2A,B). The hMSCs were subsequently induced to osteoblast- or chondrocyte-like cells and encapsulated into alginate beads of a diameter of ~1 mm. At the beginning, the cells were scattered within the alginate; after five days, the newly divided cells aggregate together and form clumps (Figure 2C,D).

### 2.2. Mineralization of the Cells, Triggered to Osteogenic Differentiation

The hMSCs were encapsulated into alginate, either free of additional components “control-alginate” or containing either enzymatically synthesized biosilica “BS-alginate” or polyP (Ca^2+^ salt) “polyP-alginate”. In our previous studies we used 100 to 400 μM prehydrolyzed TEOS (tetraethyl orthosilicate), together with silicatein [29] and polyP in the range of 50 to 100 μM [30] to activate cell metabolism and the gene expression of osteoblasts. In the present study, we exposed the hMSCs to 200 μM prehydrolyzed TEOS, together with 20 μg/mL of silicatein or to 50 μM polyP (Ca^2+^ salt).

The hMSCs were incubated as “control-alginate”, as “BS-alginate” or as “polyP-alginate” beads for 10 days in medium/serum in the absence or in the presence of osteogenic medium. This cocktail is composed of dexamethasone, ascorbic acid and sodium β-glycerophosphate [22], in order to trigger the hMSCs to form the osteogenic differentiation lineage. During the incubation of the biosilica-containing or polyP-containing beads, those polymers (biosilica or polyP) were present also in the medium at the same concentrations.

**Figure 3:**
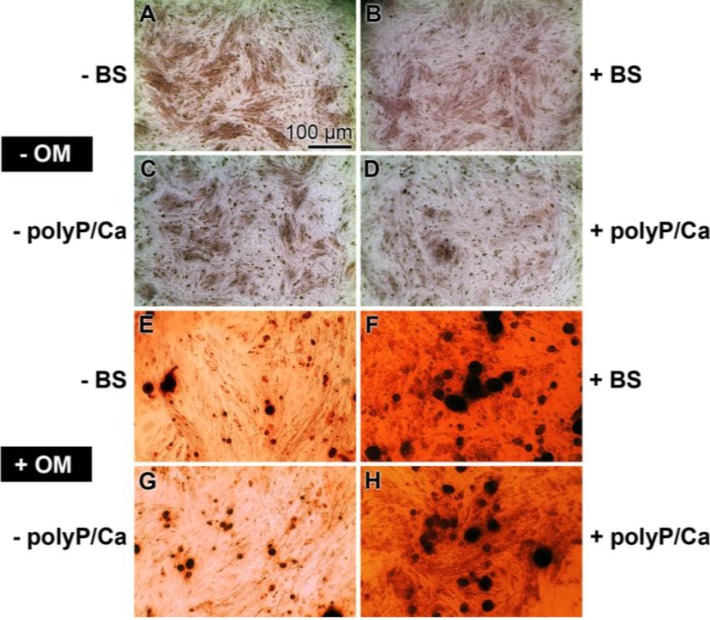
Induction of mineralization of hMSCs, embedded in alginate matrix, if incubated in the presence of osteogenic medium (OM), composed of dexamethasone, ascorbic acid and sodium β-glycerophosphate. The hMSCs were transferred to alginate beads and were incubated in the absence of osteogenic medium (−OM) or the presence of osteogenic medium (+OM) for 10 days. In two series of experiments, the cultures remained either without biosilica (−BS) or without polyP (Ca^2+^ salt) (−polyP/Ca) or were exposed to those polymers (+BS; +polyP/Ca). Subsequently, the cross-linkages of alginate matrix were partially dissolved with Na-citrate and reacted with Alizarin Red S. The cells exposed to osteogenic medium and either biosilica or polyP (Ca^2+^ salt) (+OM +BS; +OM +polyP/Ca) showed the strongest staining intensity to the dye. All images are in the same magnification; the scale is shown in (A).

After a 10-day’s incubation period, the alginate matrix was partially de-cross-linked with Na-citrate for 15 min. Then, the cells were transferred to a microscope slide and stained with Alizarin Red S. The results show that the cells, not treated with osteogenic medium, are not stained, irrespective of whether they were exposed to biosilica or to polyP or remained without these polymers (Figure 3A–D). However, the intensity of the red staining of the cells increased strongly if the cells were incubated with osteogenic medium (Figure 3E–H). The level of intensity further increased if the hMSCs were incubated, in addition to osteogenic medium (Figure 3E,G), either with biosilica or with polyP (Ca^2+^ salt) (Figure 3F,H).

### 2.3. Osteogenic *versus* Chondrogenic Differentiation: Effect of Biosilica and Polyp on *BMP-2* Expression

Earlier, we reported that both biosilica and polyP cause *BMP-2* gene induction in SaOS-2 cells, if they grow in liquid medium [22]. In the present study, using alginate-encapsulated hMSCs, we exposed the beads either to osteogenic medium, to induce osteogenic differentiation, or to chondrocyte differentiation medium, to direct hMSCs to differentiation in the chondrogenic direction. The two sets of bead cultures were kept in the absence (“control-alginate”) or the presence of either biosilica (“BS-alginate”) or polyP (Ca^2+^ salt) (“polyP-alginate”). Samples were taken after one day, five days or 10 days. The alginate matrix around the cells was solubilized, and the released cells were subjected to real-time RT (reverse transcription)-PCR (qRT-PCR) to quantitatively determine the expression level of *BMP-2*. The expression was correlated to the expression of the house-keeping gene, *GAPDH*.

**Figure 4:**
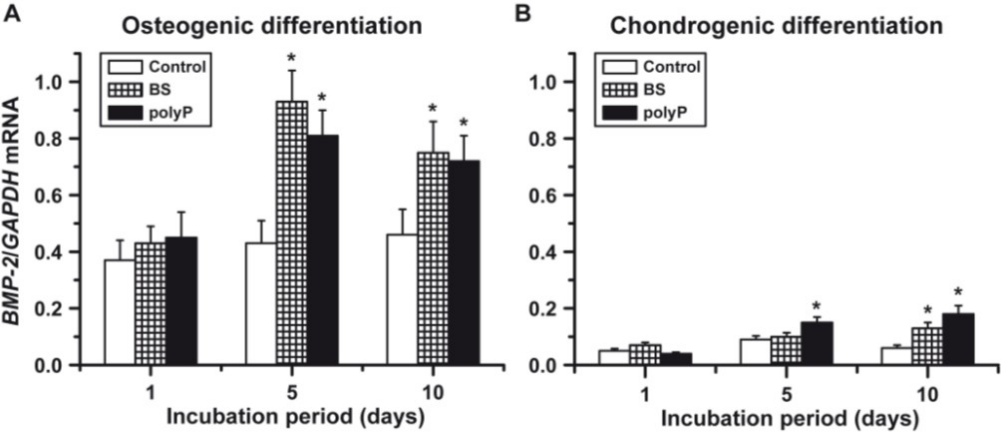
Levels of *BMP-2* transcripts in hMSCs after (**A**) induction with osteogenic medium to the osteogenic lineage or (**B**) triggering to form the chondrogenic lineage with chondrocyte differentiation medium (CDM). The cells were embedded into alginate beads and exposed to biosilica (BS; squared bars) or polyP (Ca^2+^ salt; closed bars) (polyP), as described in “Experimental Section”; the controls (open bars) did not receive those polymers (BS or polyP). Samples of beads were collected at Day 1, Day 5 and Day 10, after starting the experiments. The cells were released from the alginate matrix and, after isolation of the RNA, subjected to real-time RT (reverse transcription)-PCR (qRT-PCR). The expression level of *BMP-2* was normalized to the expression of *GAPDH*. Data are expressed as mean values ± SD for four independent experiments. Differences between the groups were evaluated using the unpaired *t*-test. * *p* < 0.05.

The experiments revealed that in the osteoblasts lineage, a high steady-state level of *BMP-2* transcription occurred already after five days if the cells in the beads were exposed either to biosilica or to polyP (Ca^2+^ salt). This increase is about two-fold, if compared to control cultures. After an extended incubation for 10 days, the transcription level drops to 1.5-fold, with respect to the controls (Figure 4A). In contrast, the expression level of BMP-2 in the chondrogenic lineage is low, and (measured in the controls) it is approximately 15% of the level seen in the osteogenic lineage. If the cells/beads are exposed for five days or 10 days to biosilica or polyP (Ca^2+^ salt), the level of *BMP-2* in the chondrogenic lineage increases only up to about 25% of the levels found in the osteogenic lineage (Figure 4B).

### 2.4. Osteogenic *versus* Chondrogenic Differentiation: Expression of *ALP*

**Figure 5:**
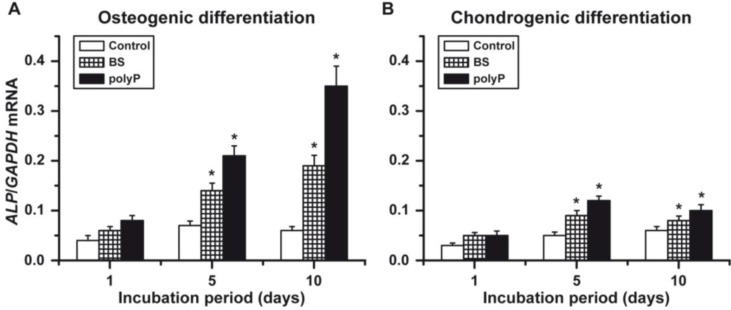
The induction *alkaline phosphatase* (*ALP*) gene in alginate-encapsulated hMSCs induced to either (**A**) the osteogenic lineage or (**B**) the chondrogenic lineage. The cells in those beads were incubated in the absence (controls; open bars), or the presence of either biosilica (BS; squared bars) or polyP (Ca^2+^ salt) (polyP; closed bars). * *p* < 0.05.

As expected, the basal level of the *ALP* expression is already about 1.4-fold higher in the osteogenic cells (Figure 5A), compared to the transcription level in the chondrogenic cells (Figure 5B). This level is significantly increased to two-fold after the five-day incubation period in the presence of biosilica or in the presence of polyP (Ca^2+^ salt) to three-fold. After an incubation period of 10 days, this increase is even enhanced; the level in the presence of polyP (Ca^2+^ salt) is 5.9-fold compared to the controls.

Furthermore, chondrocytes contain the ALP that cleaves pyrophosphate [31], even though at a lower level. The overall *ALP* transcription level in chondrogenic cells is up to about 30% lower compared to the osteogenic cells (Figure 5B). The inducing activity of both biosilica and polyP (Ca^2+^ salt) in chondrogenic cells is significant. However, compared to the osteogenic cells, the level of induction is about three-fold lower in chondrogenic cells.

### 2.5. Osteogenic *versus* Chondrogenic Differentiation: Expression of *Collagen Type I*

Osteoblasts can be differentiated in their expression levels of the collagen isoforms. While during osteogenic differentiation, the *collagen type I* gene undergoes a strong and increased expression [15,32], chondrocytes express primarily *collagen type II* [33].

**Figure 6:**
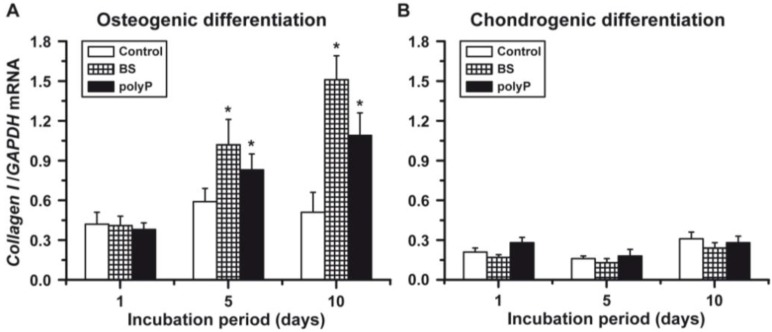
Effect of biosilica or polyP (Ca^2+^ salt) on the expression level of *collagen type I*. The osteogenic cells (**A**) or the chondrogenic cells (**B**) were incubated with biosilica, or with polyP (Ca^2+^ salt) or remained untreated (controls). * *p* < 0.05.

The steady-state expression of the *collagen type I* gene is higher in the osteogenic cells (Figure 6A), compared to the chondrogenic cells (Figure 6B). If the osteogenic cells are exposed to both biosilica and polyP (Ca^2+^ salt), an increased steady-state mRNA level is seen at Day 5 and Day 10; this increase is more pronounced in cultures supplemented with biosilica. No significant alteration of the *collagen type I* transcription level was measured in response to biosilica or polyP (Ca^2+^ salt) in chondrogenic cells (Figure 6B). 

### 2.6. Osteogenic *versus* Chondrogenic Differentiation: Expression of the *Collagen Type II*

**Figure 7:**
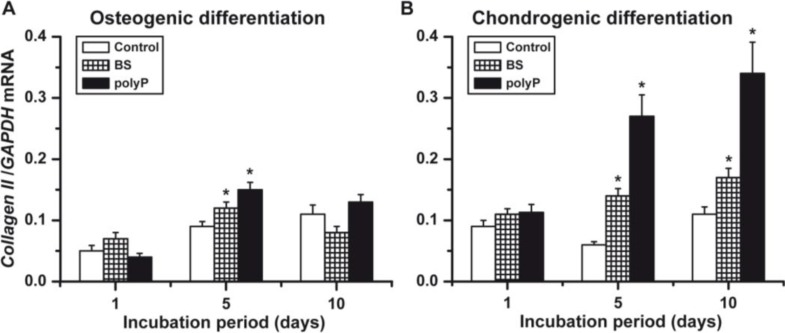
Gene expression of *collagen type II* in osteogenic cells (**A**) *versus* chondrogenic cells (**B**). As marked, the induced hMSCs were incubated with either biosilica, or polyP (Ca^2+^ salt) or remained without these polymers (controls). * *p* < 0.05.

The overall gene expression level of *collagen type II* in osteogenic cells (Figure 7A) is about half the one measured in the chondrogenic cells (Figure 7B). The inductive effect of biosilica and polyP (Ca^2+^ salt) is low in osteogenic cells, compared to the one measured for chondrogenic cells. Biosilica and polyP (Ca^2+^ salt) cause, at Day 5, a significant *collagen type II* gene induction in osteogenic cells. This increase of the steady-state expression of *collagen type II* is low in osteogenic cells compared to the expression seen for chondrogenic cells.

### 2.7. Discussion

The major challenge to realize an effective 3D printing of custom-built tissue implants is the development of a suitable morphogenetically active scaffold that allows the cells to be embedded to communicate with each other and to direct the individual cells to undergo differentiation into functional “terminally”-differentiated cells. Those cross-talking cells in a fluid/solid matrix will not only fill the space within the damaged tissue, but will also facilitate the replacement of the implanted material by physiologically developing cell assemblies, giving rise to spatially organized multicomponent tissues and structures. Alginate has been found to be a promising matrix for cells, potentially to be used for 3D printing [34]; this plant-derived polymer is, however, morphogenetically inactive. However, if alginate is enriched with organic cytokines/morphogens, e.g., BMP-2 [35], this inert matrix acquires the potency to facilitate the differentiation of stem cells *in vitro*. A further approach, which is likewise physiological, but by far less expensive, is to substitute the organic growth factors in the alginate with inorganic polymers, e.g., biosilica or polyP. Both inorganic polymers are physiologically formed in metazoans [24,36].

**Figure 8:**
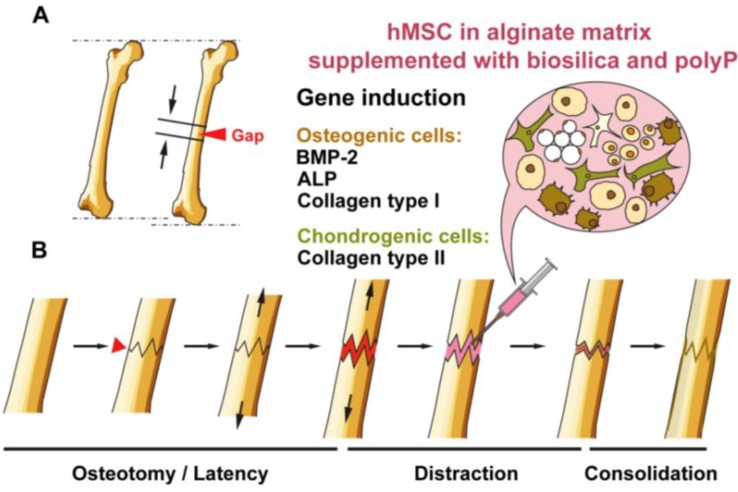
Distraction osteogenesis scheme. (**A**) The healthy part of the bone is broken into two segments with an external instrument. A distraction gap is formed; (**B**) distraction osteogenesis phases: osteotomy/latency, distraction, consolidation. During the distraction phase, hMSCs, embedded into alginate, are proposed to be injected into the fracture zone. These supplements are hoped to accelerate the velocity of bone formation under shortening the consolidation period. This alginate matrix is proposed to be supplemented with the morphogenetically active inorganic polymers, biosilica and polyP.

Besides 3D tissue printing, a morphogenetically active matrix is also needed to accelerate the regeneration step during DO. This technique, DO, requires a long time, during which the external fixator is attached at the limb during the consolidation phase. To ameliorate the medical, psychological, social and financial problems for the patient, successful attempts have been made to administer organic cytokines/morphogens, e.g., BMP-2, to accelerate bone formation [37]. During the first phase of DO/osteotomy, the latency period, distraction starts under the formation of soft callus and initiation of bone repair processes [14] (Figure 8). During the distraction phase and the subsequent consolidation period, the bone-lacking gap is filled with bone cells until the bone is solidified and the fractures are healed.

The present study shows that both biosilica and polyP provide hMSC-containing alginate hydrogels with a morphogenetic/osteogenic potential, resulting in an increased growth and differentiation potential of the osteoblasts and their precursor cells. In detail, the genes encoding BMP-2, ALP and collagen Type I are induced, three functional and structural prerequisites for a functional activity of the osteoblasts. Furthermore, BMP-2 is known to induce ectopic bone formation and increases bone repair in several animal models. BMP-2 and the related morphogens, BMP-4 and BMP-7, promote cell differentiation into osteoblasts (see [38]). ALP, for a long time already implicated in biomineralization, is a feature of the osteoblast phenotype [39]. Moreover, experimental evidence is available that ALP, which is associated with cell membranes and matrix vesicles, is involved in bio-seed formation during hydroxyapatite formation (see [40]). *Collagen type I* gene expression precedes ALP formation in osteoblasts and in a back-circuit, promotes osteoblast differentiation from precursor cells [41].

Biosilica and polyP also act in a stimulatory manner on the differentiation of hMSCs to chondrogenic cells. Both polymers have been shown to induce in hMSCs, embedded into alginate, the gene encoding for collagen Type II. This fibrillar structural protein, again in a back-circle, promotes chondrogenic differentiation from hMSCs [42].

## 3. Experimental Section

### 3.1. Isolation and Cultivation of Human MSCs

The hMSCs were isolated using previously described methods [25,43]. The human cells were obtained, after approval from the ethics committee, from bone marrow aspirations after informed consent of the donors. The deep-frozen, preserved hMSCs were thawed, and 1 × 10^6^ cells were suspended in one 75-cm^2^ flask (Cat. no. 658175; Greiner, Frickenhausen, Germany) and cultivated in α-MEM (Cat. no. F0915; Biochrom, Berlin, Germany), supplemented with 20% fetal calf serum (FCS; Gibco Invitrogen, Carlsbad, CA, USA), as well as with 0.5 mg/mL of gentamycin, 100 units penicillin and 100 μg/mL of streptomycin, as well as 1 mM pyruvate (Sigma, Taufkirchen, Germany). The characteristics of the hMSCs with respect to their osteoblast, as well as adipogenic and chondrogenic differentiation potentials, have been given in detail [25,43]. The incubation was performed in a humidified incubator at 37 °C and 5% CO_2_. After 2 days, the non-adherent cells were discarded, and the adherent cells continued to be incubated with α-MEM/FCS. Then, the culture medium was renewed every 3 days. At ~80% confluence, the cells were suspended using a 0.25% trypsin/0.02% EDTA solution (Sigma) and plated at a concentration of ~5000 cells/cm^2^. The cultures were split at a ratio of 1:3 every 5 to 6 days after the first passage. Those cells were used for the experiments, mainly for encapsulation into alginate.

The assays for staining the cells were performed in 24-well plates (Cat. no. 662160; Greiner), while the experiments with cells subjected to qRT-PCR were performed either in 48-well plates (Cat. no. 677102; Greiner) or in 25-cm^2^ flasks (Cat. no. 690175; Greiner). At the time of harvesting the cells, the cultures were approximately 80% confluent.

### 3.2. Preparation of Alginate/Silica and Alginate/PolyP (Ca^2+^ Salt) Composite Hydrogel Beads

Adherent cells from ~80% confluent cultures were released from the plates and suspended in 1.2% (w/v) alginate (Na-alginate, Cat. no. CAA W20,150-2, low viscosity, dissolved in PBS, pH 7.4; Sigma) and processed as described [34]. In brief, the cell-hydrogel suspensions were passed through a needle (dimension: 0.45 × 6 × 23 mm) attached to a 1 mL syringe and dropped into a 1.5% (w/v) CaCl_2_ solution. After 5 min, the approximately 1 mm-large beads were washed three times in saline and then twice in α-MEM medium. The beads (approximately 60 beads/well) were placed into 24-well plates (Nunc, Langenselbold, Germany) and incubated in 3 mL of α-MEM/FCS in the presence of the respective induction medium. Those cultures were termed “control-alginate”.

After termination of the experiments, the cells were released from the beads by washing twice with PBS and transferring them into 2 mL of 55 mM Na-citrate; after 30 min at 37 °C, the cells were collected by centrifugation (900× *g*, 5 min; [34,44]) and used for quantitative real-time RT-PCR determinations. For staining the cells with Alizarin Red S, the period for de-cross-linking of alginate with Na-citrate was shortened to 15 min. Then, the cells were stained.

Where indicated, the alginate beads were supplemented with biosilica or with polyP. Biosilica was prepared as described [29]. Prehydrolyzed TEOS (tetraethyl orthosilicate; Sigma) in a concentration of 200 μM was added to 20 μg/mL of recombinant silicatein; these components were added to the alginate matrix. After each medium change, prehydrolyzed TEOS/recombinant silicatein was added again to the medium. The biosilica-containing beads were termed “BS-alginate”. In a control experiment, silicatein was replaced in the assays by 20 μg/mL of bovine serum albumin (BSA); those beads displayed no effect on the expression of the genes selected in the present study (data not shown). The beads were incubated for up to 10 days.

Na-polyP (average chain of approximately 40 phosphate units) was obtained from Chemische Fabrik Budenheim (Budenheim, Germany). To compensate for any effect, caused by a potential chelating activity of polyP to Ca^2+^, the polymer was mixed together with CaCl_2_ in a stoichiometric ratio of 2:1 (polyP:CaCl_2_), as described [30]; the salt, designated as “polyP (Ca^2+^ salt)”, was added to the beads and the medium at a concentration of 50 μM. Incubation was again for up to 10 days. The beads containing polyP were termed “polyP-alginate”.

### 3.3. Differentiation Assays *in Vitro*

Osteogenic differentiation of hMSCs, encapsulated into hydrogel beads, was performed by exposure of the cells to the osteogenic medium, as described [22]. Osteogenic medium contained in the α-MEM/FCS, dexamethasone (Sigma), ascorbic acid (Sigma) and sodium β-glycerophosphate (Sigma). As end-point marker for osteoblasts, the extent of mineralization based on Alizarin Red S staining, as well as the gene expression levels of selected marker genes was determined.

Chondrogenic differentiation of the stem cells was induced in α-MEM/FCS supplemented with chondrocyte differentiation medium, as described [3,27]. It consists of premix tissue culture supplement (Becton Dickinson, Heidelberg, Germany), dexamethasone, ascorbate-2-phosphate, pyruvate and transforming growth factor-β1 (Sigma), human insulin (Sigma) and transferrin (Sigma).

The described experiments were performed after having transferred the hMSCs to the respective activation medium.

The cells/beads were inspected with light microscopically using either a Keyence BZ-8000 epifluorescence microscope or a Keyence VK-8710K, color 3D laser microscope (Neu-Isenburg, Germany).

### 3.4. Mineralization Assay with Alizarin Red S

Mineralization by differentiated hMSCs was qualitatively assessed by staining the cell cultures on the coverslips with 10% Alizarin Red S, after fixation with ethanol [45].

### 3.5. Quantitative Real-Time RT-PCR (qRT-PCR) Analysis

The technique of quantitative real-time RT (reverse transcription)-PCR (qRT-PCR) was applied to determine the levels of transcription of the following genes: *BMP-2* (*bone morphogenetic protein-2*; NM_001200.2) Fwd: 5′-ACCCTTTGTACGTGGACTTC-3′ (nt_1681_ to nt_1700_); and Rev: 5′-GTGGAGTTCAGATGATCAGC-3′ (nt_1785_ to nt_1804_; 124 bp); *ALP* (*alkaline phosphatase*; NM_000478.4) Fwd: 5′-TGCAGTACGAGCTGAACAGGAACA-3′ (nt_1141_ to nt_1164_); and Rev: 5′-TCCACCAAATGTGAAGACGTGGGA-3′ (nt_1418_ to nt_1395_; 278 bp); *COLI* (*collagen type I*; NM_000088) Fwd: 5′-TATGGGACCCCAAGGACCAAAAGG-3′ (nt_1122_ to nt_1145_); and Rev: 5′-TTTTCCATCTGACCCAGGGGAACC-3′ (nt_1234_ to nt_1257_) (136 bp); *COLII* (*collagen type II*, alpha 1 (COL2A1), transcript variant 1, mRNA: NM_001844) Fwd: 5′-TCCATTCATCCCACCCTCTCAC-3′ (nt_4755_ to nt_4776_); and Rev: 5′-TTTCCTGCCTCTGCCTTGACC-3′ (nt_4902_ to nt_4882_; 148 bp). As a reference gene, GAPDH (glyceraldehyde 3-phosphate dehydrogenase; NM_002046.3) (Fwd: 5′-CCGTCTAGAAAAACCTGCC-3′ (nt_845_ to nt_863_); and Rev: 5′-GCCAAATTCGTTGTCATACC-3′ (nt_1059_ to nt_1078_; 215 bp)) was used.

The cells were released from the hydrogel samples and collected by centrifugation. After RNA extraction using the TRIzol reagent (Invitrogen GmbH, Darmstadt, Germany), the samples were subjected to qRT-PCR. For that, 2 μL of the appropriate dilution were employed as a template in the 30 μL qRT-PCR assays. All reactions were run with an initial denaturation at 95 °C for 3 min, followed by 40 cycles, each with 95 °C for 20 s, 58 °C for 20 s, 72 °C for 20 s and 80 °C for 20 s. Fluorescence data were collected at the 80 °C step. The runs were performed in an iCycler (Bio-Rad, Hercules, CA, USA). The mean *C*_t_ values and efficiencies were calculated by applying the iCycler software (Bio-Rad); the estimated PCR efficiencies were in the range of 93%–103%. Expression levels were correlated to the GAPDH reference gene to determine relative expression, as described [21].

### 3.6. Further Analyses

The results were statistically evaluated using the paired Student’s *t*-test [46]. DNA content was determined by application of the PicoGreen method, as described [45], using calf thymus DNA as a standard. 

## 4. Conclusion

In conclusion, for the two inorganic polymers, biosilica and polyP, which are abundantly found in marine organisms, our findings extend our earlier results obtained with (almost) terminally differentiated osteoblasts (reviewed in [17]), establishing that, both inorganic polymers, biosilica and polyP, are potent morphogenetically active additives of the alginate matrix.
